# IR64: a high-quality and high-yielding mega variety

**DOI:** 10.1186/s12284-018-0208-3

**Published:** 2018-04-09

**Authors:** David J. Mackill, Gurdev S. Khush

**Affiliations:** 1 Mars, Inc. and Department of Plant Sciences, University of California, Davis, CA 95616 USA; 2 Department of Plant Sciences, University of California, Davis, CA 95616 USA

## Abstract

High-yielding varieties developed in the 1960s and 1970s at the International Rice Research Institute (IRRI) and elsewhere benefited farmers and the public, ultimately increasing yields and reducing the cost of rice to consumers. Most of these varieties, however, did not have the optimum cooking quality that was possessed by many of the traditional varieties they replaced. In 1985, the IRRI-developed *indica* variety IR64 was released in the Philippines. In addition to its high yield, early maturity and disease resistance, it had excellent cooking quality, matching that of the best varieties available. These merits resulted in its rapid spread and cultivation on over 10 million ha in the two decades after it was released. It has intermediate amylose content and gelatinization temperature, and good taste. It is resistant to blast and bacterial blight diseases, and to brown planthopper. Because of its success as a variety, it has been used extensively in scientific studies and has been well-characterized genetically. Many valuable genes have been introduced into IR64 through backcross breeding and it has been used in thousands of crosses. Its area of cultivation has declined in the past 10 years, but it has been replaced by a new generation of high-quality varieties that are mostly its progeny or relatives. Continued basic studies on IR64 and related varieties should help in unraveling the complex genetic control of yield and other desirable traits that are prized by rice farmers and consumers.

## Background

In November 2016, the International Rice Research Institute (IRRI) and others commemorated the 50th anniversary of the release of IR8, its first developed variety and a beginning of the Green Revolution in rice (http://irri.org/ir8). This variety established the basic plant type of the high-yielding varieties (HYVs) that have now spread over most rice growing areas.

IR8 had a very high grain yield, but also a number of defects, most importantly, poor grain quality, lack of disease and insect resistance, and late maturity. The varieties subsequently developed and released over the next two decades improved greatly on these traits (Khush 1999). During the early 1980s, one of the most popular varieties grown was IR36. In addition to its disease and insect resistance, it achieved its high yield in a period of only 111 days from seed to seed, compared to 130 days for IR8 (Khush and Virk 2005). It spread rapidly and was estimated to be planted on more than 10 million ha during the 1980s.

While much improved over IR8, IR36 still lacked the quality of the best varieties grown in the Philippines and Indonesia before the Green Revolution. IR64, released in the Philippines in 1985, represented a breakthrough in combining excellent palatability of cooked rice with the other traits found in previous IRRI HYVs. IR64 replaced IR36 in most growing areas and spread rapidly in new areas. Because of its wide adaptation, early maturity, and improved quality, it became a standard for high-quality rice and was highly desired by the rice industry. Because of its popularity, it has been used widely as a representative *indica* variety in research studies. It has also been used extensively as a parent in breeding programs, and to develop populations for genetic analysis.

In this article, we describe the development of IR64 and its major characteristics. We also discuss the use of this variety in further breeding and rice research.

## Review

### Breeding history, including parentage and selection history, evaluation and release

The breeding history of IR64 is summarized in Khush and Virk (2005). The breeding program at IRRI focused on combining the different traits desired by farmers, including high yield, resistance to biotic and abiotic stresses, early maturity, and improved grain quality. The Genetic Evaluation and Utilization (GEU) program was formulated at IRRI to focus research efforts on combining these traits through interdisciplinary collaboration (Khush and Coffman 1977). In segregating generations and in evaluation of fixed lines, plants and breeding lines were evaluated for these traits either in the pedigree breeding nursery or special screening nurseries. This improved the chance of combining multiple traits into a single variety.

**Fig. 1:**
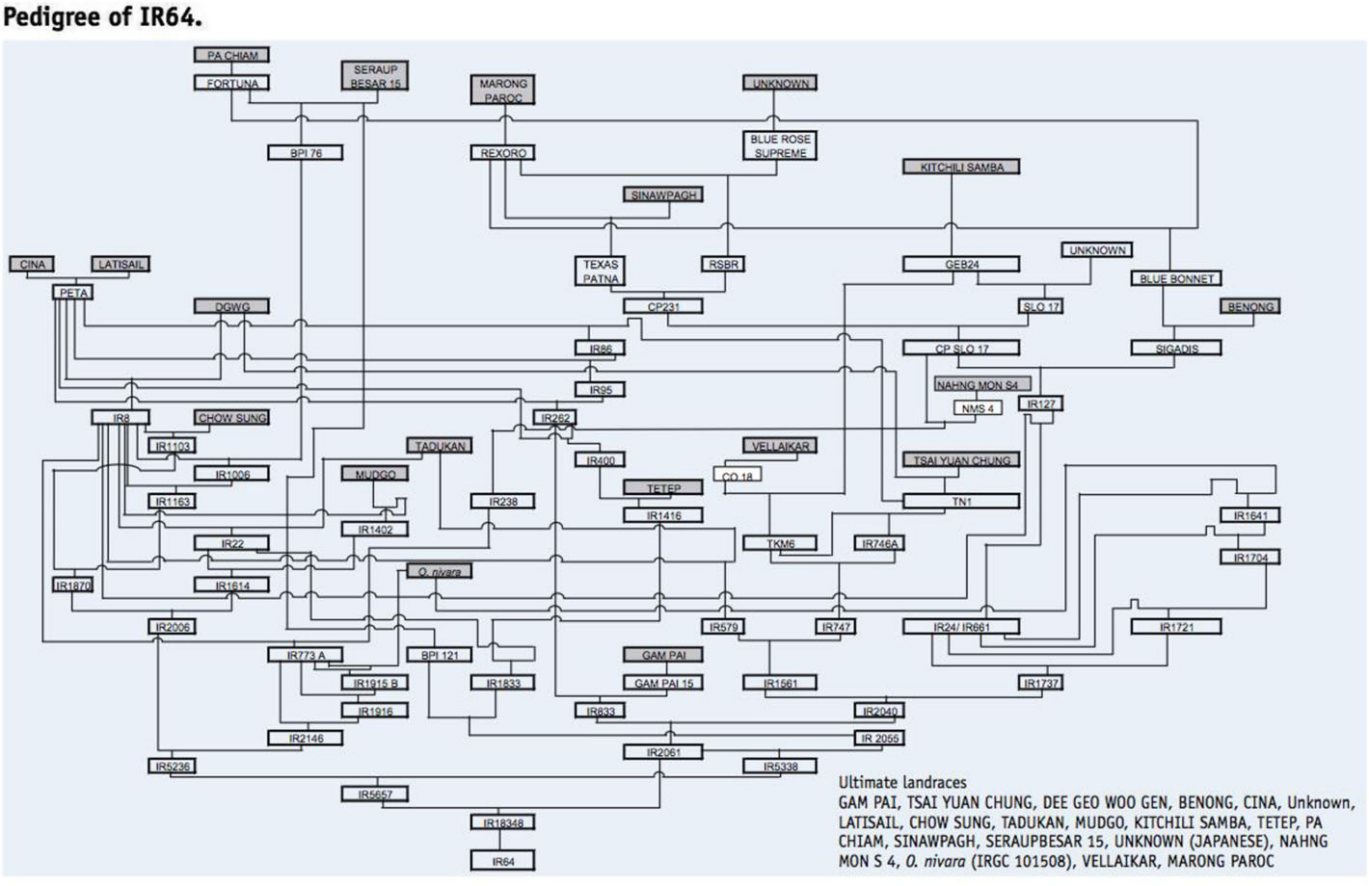
Pedigree of IR64 showing the ultimate landraces in its ancestry (Khush and Virk 2005)

The cross of IR5657-33-2-1/IR2061-465-1-5-5, was made in early 1977 and was designated IR18348. The female parent was noted for its good cooking quality (intermediate amylose), as well as having some tolerance to salinity. The male parent was a high-yielding breeding line derived from a highly productive cross that resulted in a number of other varieties from sister lines, including IR28, IR29 and IR34. The full pedigree (Fig. 1) shows the derivation of IR64 from 19 traditional rice varieties.

The F2 population was evaluated in 1978, and the pedigree method of breeding was followed in the F3 and F4 populations, grown in 1979. The breeding line IR18348-36-3-3 resulted from the bulk harvest of a F_5_ family in 1980, and was subsequently evaluated in yield trials in 1981–83 at IRRI, as well as in the Philippine National Trials. It out-yielded IR36 by 21% in these trials (IRRI 1986). It was released by the Philippine Seed Board with the designation ‘IR64’ in 1985, and this designation has been subsequently used by IRRI (IRRI 1986).

The early varieties developed at IRRI were high yielding and had disease and insect resistance, but their grain quality was inferior to the best available varieties. At the time, the standard for grain quality in the Philippines was the variety BPI-76 and its sister line BPI-121, derived from the cross Fortuna/Seraup Besar 15 (Cada and Escuro 1972). The parent variety Fortuna is from the USA, and Seraup Besar 15 was originally introduced from Malaysia. BPI-76 was released in 1960 in the Philippines, and non-photoperiod sensitive selections, BPI-76 (n.s.) and BPI-76-1, were also released (Dalrymple 1978). Another popular variety noted for its quality was C4-63, and it was derived from the cross Peta/BPI-76. Before IR64, C4-63 was considered one of the main high-quality varieties in the Philippines. In 1970, a green-base C4-63 was released to replace the original seed stocks of C4-63. Because of its good eating quality (intermediate amylose content) it had spread in Indonesia, Malaysia, and Burma (Yoshida 1981). Rice was even widely marketed under the C4 name up to the 1980s; however, many of the market samples actually turned out to be other varieties or mixtures of varieties with inferior quality that were being grown by farmers (Juliano et al. 1989).

BPI-121-407 is considered the most likely contributor of superior quality in the pedigree of IR64, although BPI-76 is also in its pedigree. BPI-121-407 is a short-statured breeding line with superior quality, and was selected as an induced mutant of the original BPI-121 (Cada and Escuro 1972). In the advanced evaluation of the breeding line IR18348-36-3-3 at IRRI, taste panels were used to ensure that the quality of BPI-76 (or BPI-121) was captured. Taste panels were also applied in the government of the Philippines rice program to assist in providing the data for release of this variety.

### Adoption and impact

The major breakthrough in the development of IR64 was the combination of the high yield and disease and insect resistance of earlier IRRI varieties with the superior grain quality associated with varieties like BPI-76 and C4-63. It’s first release was by the Philippine government in 1985. It was also released in the following countries: Bhutan, Burkina Faso (as FKR42), Cambodia, China, Ecuador (as NIAP11), Gambia, India, Indonesia, Mauritania, Mozambique, and Vietnam (as OM89) (Khush and Virk 2005). Its wide adaptation is notable, and it became widely grown in Southeast and South Asia. It is also noted to be well adapted to the Sahelian regions of West Africa (Devries et al. 2011; Julia and Dingkuhn 2013).

**Fig. 2:**
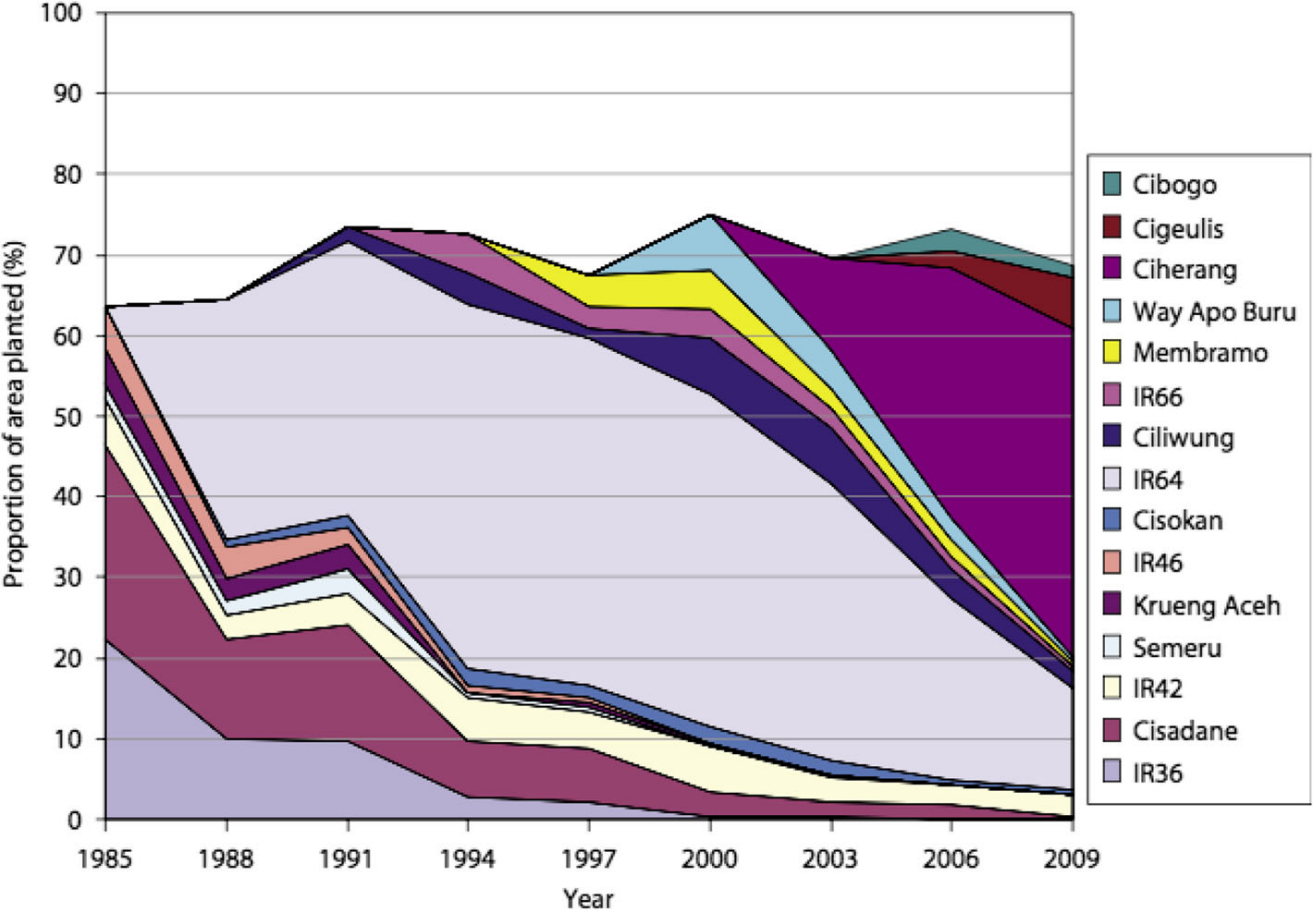
Share of leading varieties in Indonesia during 1985–2009 (Brennan and Malabayabas 2011)

By 1995, IR64 was already estimated to be grown on 8 million ha (Khush 1995), and by the turn of the century this rose to over 10 million ha. The long persistence of IR64 in farmers’ fields after its release was attributed to its excellent eating quality (Champagne et al. 2010). The area of production gradually declined in the Philippines during the period 2000–2007, partly due to pressure from tungro disease (Laborte et al. 2015). Indonesia was a major producer of IR64, which was grown on more than 40% of its total area for around a decade (Fig. 2), and was still popular in 2009 (Brennan and Malabayabas 2011). It is also widely grown in India. During 1998–2006 IR64 accounted for over 10% of the breeder seed produced in India and was still above 3% in 2015, suggesting that it was grown on 2–3 million ha annually during the period (data provided by A. K. Singh).

A specific estimate of the impact of this variety has not been attempted, even though it is known as the most popular variety in terms of area, particularly in tropical Asia. IR64 has contributed greatly to farmer incomes not only through higher yields, but through improved quality that results in higher price and earlier maturity that allows higher cropping intensity.

As with other IRRI varieties, seed of IR64 was distributed freely to researchers and farmers and no intellectual property protection was sought on it or any progeny developed from it.

### Major characteristics, including key traits

**Fig. 3:**
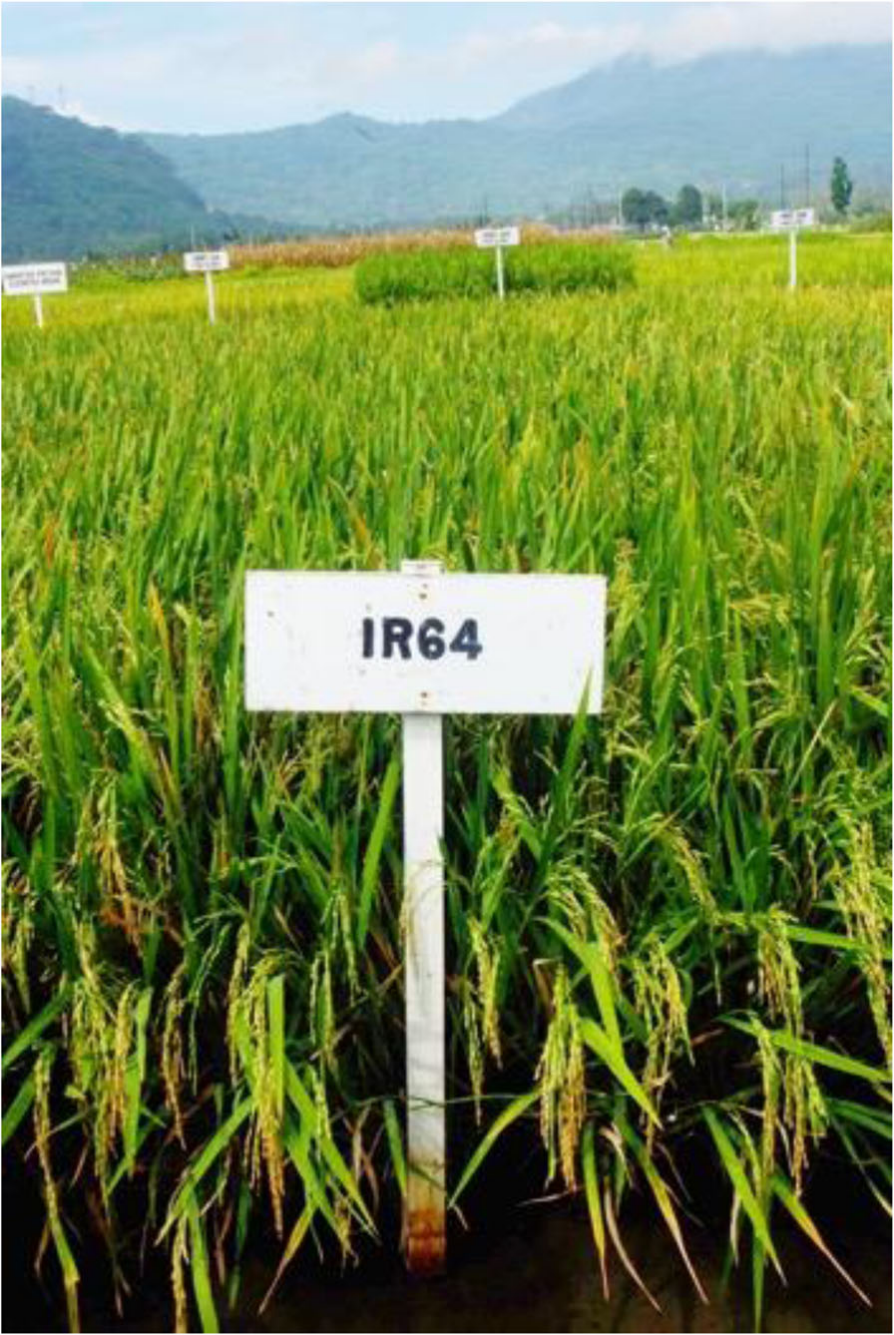
Plot of IR64 growing in the field at IRRI, Los Baños, Philippines (photo from IRRI)

IR64 is a semidwarf *indica* rice variety, with average mature plant height of approximately 100 cm in the Philippines (Fig. 3). It is a relatively early duration variety, with total growth duration of about 117 days (Khush and Virk 2005). It inherits the same semidwarf *sd1* allele as other IRRI semidwarf varieties, ultimately derived from Dee-geo-woo-gen. According to Wei et al. (2016) it has the loss of function alleles for *Hd1* and *Ehd1*, which confer earlier duration and insensitivity to photoperiod. At the time of its release, IRRI (1986) listed the valuable traits as resistance to brown planthopper (BPH) biotypes 1 and 3, green leafhopper (GLH), white backed planthopper (WBPH), bacterial blight, grassy stunt virus; and moderate resistance to blast, BPH biotype 2, and stem borer. It was the first IRRI variety to combine intermediate amylose content and intermediate gelatinization temperature (GT).

IR64 has high yield, especially compared to earlier-released IRRI varieties, but not as high as some of the subsequently released varieties like IR72. Peng et al. (2000) list its grain yield as 8.76 and 8.28 t ha^− 1^ compared to 9.50 and 9.06 t ha^− 1^ for IR72 in the dry season at IRRI in 1996 and 1998, respectively. It has high grain filling percentage and grain weight, but a relatively low spikelet number per m^2^ (Peng et al. 2000). According to Ujiie et al. (2016), IR64 possesses a gene *GS3* for grain size and the narrow-leaf gene (*NAL1*) that improve grain yields. IR64 is considered a typical high-tillering *indica* type cultivar, in contrast to the “new plant type” NPT varieties that have lower tillering but large tiller and panicle size (Okami et al. 2015).

IR64 has relatively durable resistance to BPH, and it is known to carry the major gene *Bph1*. However, it is reported to have better resistance than other varieties carrying *Bph1* and has good field resistance to the pest, exhibiting antiobiosis, antixenosis and tolerance (Cohen et al. 1997). This is partly attributed to its possessing additional QTLs controlling BPH resistance which confer greater durability of the resistance (Alam and Cohen 1998).

IR64 has very good levels of resistance to blast disease, including major-gene resistance and partial resistance (Bastiaans and Roumen 1993; Grand et al. 2012; Roumen 1992). Sallaud et al. (2003) reported six resistance genes, designated *Pi25(t), Pi-27(t), Pi29(t), Pi30(t), Pi31(t),* and *Pi32(t).* It also has resistance genes *Pita* (Lee et al. 2011) and *Pi20* (Khush and Virk 2005), as well as *Pi33* on chromosome 8 which was derived from *O. rufipogon* (accession IRGC101508, sometimes referred to as *O. nivara*) in its pedigree (Ballini et al. 2007; Berruyer et al. 2003). Sreewongchai et al. (2010) found that IR64 has broad resistance to blast disease in Thailand and this was conferred mainly by QTLs on chromosomes 2 and 12. It was also resistant in a blast hotspot in India (Thakur et al. 2013). Kongprakhon et al. (2010) identified QTLs that may correspond to *Pi25* (chromosome 2), *Pi29* (chromosome 8), and *Pi28* (chromosome 10).

IR64 is resistant to Bacterial Blight (BB) disease (caused by *Xanthomonas oryzae* pv. *oryzae*) and possesses the major gene *Xa4* for resistance (Adhikari et al. 1994; Khush and Virk 2005). The gene is thought to confer additional agronomic benefits in addition to BB resistance (Hu et al. 2017). It is also resistant against African strains of *X. oryzae*, and several QTLs for resistance were identified (Djedatin et al. 2016).

IR64 is susceptible to tungro disease, including Rice Tungro Spherical Virus (RTSV) (Lee et al. 2010) and Rice Tungro Bacilliform Virus (RTBV) (Zenna et al. 2006).

IR64 was developed primarily for irrigated rice production, and abiotic stress resistance was not an objective. While it is generally considered susceptible to abiotic stresses, it has been widely grown in more favorable rainfed situations and under mildly unfavorable soils.

IR64 is considered susceptible to drought stress and yield reductions can be considerable (Anantha et al. 2016). Yields under aerobic conditions (favorable upland) are also relatively low (Zhao et al. 2010). Vikram et al. (2015) attributed drought susceptibility of many modern high-yielding varieties to linkage between a drought susceptibility QTL and the semidwarf gene *sd1*. IR64 has a relatively shallow root system and low root length density (Henry et al. 2011; Shrestha et al. 2014). Under water stress, IR64 has relatively low water uptake rate (Gowda et al. 2012).

IR64 is considered sensitive to heat (Coast et al. 2016; Gonzalez-Schain et al. 2016; Shanmugavadivel et al. 2017; Ye et al. 2015), although this has not been reported as a problem with previous or current production environments. However, Jagadish et al. (2008) indicated that IR64 is actually moderately tolerant of high temperature at flowering. It is also susceptible to Fe toxicity (Wu et al. 1997), anaerobic germination (Miro and Ismail 2013), and low temperature at the vegetative stage (Chawade et al. 2013). IR64 lacks the P deficiency gene *PSTOL1* like a number of other high-yielding varieties (Gamuyao et al. 2012). IR64 was reported to be sensitive to low P conditions (Mori et al. 2016; Vejchasarn et al. 2016). It is also relatively sensitive to Zn deficiency (Impa et al. 2013). While IR64 does not possess the submergence tolerance allele of *SUB1*, it has some moderate tolerance to submergence during the vegetative stage (Singh et al. 2010; Singh et al. 2013).

### Grain and market characteristics

IR64 grain has good physical appearance and is a typical long-grain variety with high head rice yield (IRRI 1986). As mentioned above, IR64 was the first IRRI variety to have both intermediate amylose content and intermediate GT. These traits are considered important for the ideal texture of cooked rice, especially for many rice consumers in South and Southeast Asia. However, these two traits alone do not account for the superior cooking quality of the variety, and methods to evaluate cooking quality are still inadequate aside from laborious sensory methods (Concepcion et al. 2015). This is why the use of taste panels was essential to identify the superior quality of IR64.

Rice texture is mostly controlled by the allele at the *waxy* locus (*Wx*) on chromosome 6. Among the major alleles at this locus, IR64 carries the *Wx*^*in*^ allele at the *waxy* locus signifying intermediate amylose content (Zhang et al. 2012). However, various sequence features of the *Wx* gene are associated with grain quality of rice, including the number of CT repeats in the 5′ untranslated part of the gene and the SNPs at specific sites in intron 1, exon 6, and exon 10 (Bligh et al. 1998; Bligh et al. 1995; Larkin and Park 2003). Based on the DNA sequence, IR64 has the (CT)_17_ allele of *Wx* (Roferos et al. 2008; Teng et al. 2012). (CT)_17_ and (CT)_20_ are associated with intermediate amylose and soft to medium hardness (Roferos et al. 2008). The SNPs at the *Wx* locus are G-C-C for intron 1, exon 6 and exon 10, a common haplotype for intermediate amylose varieties (Chen et al. 2010).

Azucena and IR64 both have intermediate amylose and intermediate GT, and the doubled haploid population of the cross between the two was used to identify QTLs for several grain quality related traits, including starch properties measured by Rapid Visco Analyzer (RVA) (Bao et al. 2002). The study showed that there are genetic differences for these traits despite both varieties having similar amylose content and GT.

Part of the improved quality superiority is from improved flavor, including sweet and corn notes (Calingacion et al. 2015; Champagne et al. 2010). Reduced yellow color, improved texture and mouthfeel, and superior “sweet” taste were noted in IR64 vs. the lower-quality IRRI-132 (Champagne et al. 2010) (Table 1). Metabolomics analysis showed very different profiles for IR64 vs. the lower quality variety Apo (Calingacion et al. 2015).

**Table 1:** Comparison of flavor attributes of high quality IR64 and low-quality Apo (Calingacion et al. 2015)

Flavor	Apo^a^	IR64^a^	Apo		IR64	
			Irrigated	Drought	Irrigated	Drought
Sweet taste	+	++	+	+	++	+
Corn		+			+	+
Sweet aromatic		+			+	+
Astringent	++	+	+	+		+
Water like metallic	++	+	++	++		+
Sewer/animal	++		++	++		
Sour/silage	++		+	+		+
Hay-like musty			++	++	++	+

^a^ reported from a previous study (Champagne et al. 2010)

Calingacion et al. (2014) surveyed grain quality preferences in different countries and regions based on the most popular varieties in each area. However, two countries where IR64 dominated have different preferences (Indonesia and Philippines). These preferences can change over time.

### Genetic and genomic characteristics

IR64 has been used extensively in genetic studies of rice, mainly because it represents a high-yielding and high-quality *indica* variety that is widely adapted to tropical lowland growing conditions. The most well-known mapping population was a doubled haploid population of about 146 lines derived from the cross IR64/Azucena (Guiderdoni et al. 1992), and first used by Huang et al. (1997) to map important agronomic traits and by Wu et al. (1997) to map tolerance to Fe toxicity. Other mapping populations with IR64 as a parent have been developed using both *indica* and *japonica* parents. A recombinant inbred line (RIL) population of 171 families was developed in a cross between IR64 and the wild relative *O. rufipogon* and tolerance to Al toxicity was mapped (Nguyen et al. 2003). Reciprocal chromosome segment substitution lines (CSSL) were also developed in IR64 and Koshihikari backgrounds to study the inheritance of grain shape (Nagata et al. 2015).

The original genome sequence of rice used the *japonica* variety Nipponbare (IRGSP 2005). In the first report of resequencing multiple varieties, IR64 was used among 20 diverse varieties, although this only included 100 Mb of the unique fraction of the genome (McNally et al. 2009). Schatz et al. (2014) reported whole genome de novo assembly for IR64 as well as the *japonica* variety Nipponbare and the *aus* variety DJ123. The genome coverage was 88.5% and included 37,758 genes. IR64 was estimated to have 381 genes not present in either of the other two varieties, and DJ123 and Nipponbare had 297 and 786 genes not found in the other varieties. Jain et al. (2014) also reported whole genome sequencing of IR64 along with Pokkali and N22, achieving 84.5% coverage. Methylation pattern has also been studied in IR64 and compared with a *japonica* variety Dianjingyou1 and two wild ancestors (Li et al. 2012). Gene expression and identification of functional roles of genes have been carried out with this model variety, for example drought responsive genes (Ray et al. 2011), gene expression changes during different stages of development (Sharma et al. 2012), and salinity tolerance (Wang et al. 2016).

### Important progeny

The excellent grain quality of IR64 has become the standard for rice quality requirements in a number of countries. Because of its popularity with farmers, IR64 has been used widely as a parent in rice breeding, as a recipient of new genes through marker-assisted backcrossing and genetic transformation, and as a standard check for basic studies by many rice researchers. IR64 figured prominently in the mapping of many QTLs when genome-wide markers became available.

In the Philippines, IR64 was replaced by newer varieties in the early 2000s mainly due to its susceptibility to tungro disease. However, the breeders have attempted to retain the quality traits of IR64. PSB Rc82 is an example of a variety that became very popular, and IR64 is one of its grandparents. In India as well, the variety MTU 1010 became very popular, and it is a cross of Krishnaveni/IR64.

IR64 was a dominant variety in Indonesia for over two decades. In the last 10 years, it has been replaced by the variety Ciherang, which has very similar grain quality and improved yields. This variety is from the cross IR18349-53-1-3-1-3/IR19661-131-3-1//IR19661-131-3-1/IR64/IR64, and has high genetic similarity with IR64 (IRRI 2015; Septiningsih et al. 2014). It has very similar grain quality to IR64 and is also morphologically and genetically similar (Muhamad et al. 2017).

The availability of genome-wide molecular markers for marker assisted selection enabled the transfer of important traits into popular varieties like IR64 through marker-assisted backcrossing (MABC) (Collard and Mackill 2008). Early examples of this included varieties developed by pyramiding BB resistance genes into IR64, including Angke and Conde in Indonesia, and NSIC Rc142 in the Philippines (Verdier et al. 2012).

**Fig. 4:**
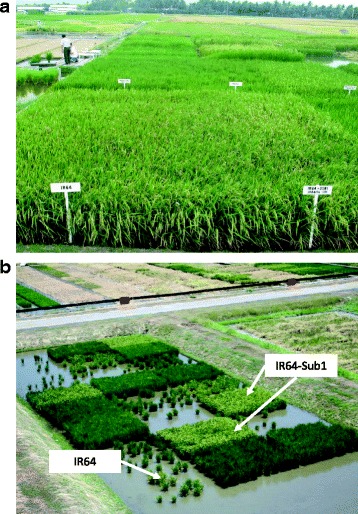
**a** IR64 and IR64-Sub1 under non-flooded conditions at IRRI. **b** Trials after submergence showing the survival of IR64-Sub1 compared to IR64. Fields were submerged for 17 days at 28 days after seeding. (Photos from IRRI)

The rice submergence tolerance gene *SUB1* was introduced by marker assisted backcrossing into IR64 and several other popular varieties (Septiningsih et al. 2009) (Fig. 4). Because IR64 had moderate tolerance to submergence, IR64-Sub1 tended to perform better under submergence than some of the other Sub1 lines, and its relatively early maturity allowed it to recover and produce yields after prolonged flooding and before the onset of low temperatures (Singh et al. 2010; Singh et al. 2013). IR64-Sub1 was released as Submarino 1 in the Philippines in 2009. The genetic similarity of Ciherang with IR64 was exploited to develop a submergence-tolerant version of Ciherang with only one backcross (Septiningsih et al. 2014). IR64-Sub1 was also used to develop submergence tolerant varieties in Vietnam (Lang et al. 2015).

Many interesting genes and QTLs have been backcrossed into IR64 and these progeny are being evaluated for useful traits (Table 2). One of the most important is drought tolerance. This trait was introduced by backcrossing from drought tolerance donor Aday Sel into IR64 (Venuprasad et al. 2011), and some of the derived lines showed yield increases of 528 to 1875 kg ha-1 over IR64 under severe drought conditions (Swamy et al. 2013). Breeding lines with two drought-tolerance QTLs (*qDTY2.2* + *qDTY4.1*) introgressed into IR64 showed improved performance under drought stress. These lines had improved hydraulic conductivity and higher root length density (Henry et al. 2015). The gene *DRO1*, conferring deeper rooting and drought tolerance, was also transferred into IR64 by backcrossing, and resulted in NILs with higher drought tolerance (Uga et al. 2013). According to the authors, a single bp deletion in DRO1 resulted in a stop codon in IR64 and caused shallow rooting and drought intolerance, and this deletion was observed in IR64 and some of its progeny but not in its ancestors. The IR64 Dro1-NIL had a 14% higher yield than IR64 (Deshmukh et al. 2017). IR64-Sub1 is being used as a recipient for drought tolerance QTLs to develop a version of IR64 tolerant of both stresses (Singh et al. 2016).

**Table 2:** Near Isogenic Lines (NILs) developed in IR64 genetic background with genes conferring novel and improved traits

Trait/QTL	Comments	References
Submergence tolerance (*SUB1*)	The *SUB1* major gene was introduced by Marker Assisted Backcrossing (MABC) into IR64 and released in several countries.	(Septiningsih et al. 2009)
Drought tolerance (*DRO1*)	Breeding line developed by MABC showed improved drought tolerance through deeper root system.	(Uga et al. 2013)
Drought tolerance (qDTY2.2 + qDTY4.1)	Lines derived by MABC showed improved yield under severe drought stress.	(Swamy et al. 2013)
SPIKE gene (NARROW LEAF1)	NIL with this gene showed 15–36% higher yield when introgressed into IR64. The gene increases spikelet number.	(Fujita et al. 2013).
Improved agronomic traits	334 introgression lines developed in IR64 background using tropical *japonica* donors	(Farooq et al. 2010; Fujita et al. 2009; Kato et al. 2010; Tagle et al. 2016)
Anaerobic germination (AG1)	IR64-AG1 was developed by introgressing the AG1 QTL into IR64.	(Toledo et al. 2015)
Yield QTL identified from *O. rufipogon*	Some QTLs from low yielding wild rice *O. rufipogon* can increase yield in IR64 background.	(Cheema et al. 2008; Septiningsih et al. 2003)
Drought tolerance from *O. glaberrima*	A population of alien introgression lines using an accession of African rice *O. glaberrima* backcrossed to IR64 (BC2), and identified QTLs associated with drought-related traits.	(Bimpong et al. 2011)
Early-morning flowering (*qEMF3*)	NIL IR64 + *qEMF3* with early morning flowering was developed using three backcrosses by marker assisted backcrossing and it flowered 1.5–2.0 h earlier in the day than IR64. In this case the donor was wild rice *O. officinalis*. This trait can confer tolerance to high temperature at anthesis.	(Hirabayashi et al. 2015)
Tolerance to P deficiency (*Pup1*)	Tolerance of P deficiency was introduced into IR64-Pup1, with the *Pup1* gene for more efficient P uptake.	(Chin et al. 2011; Wissuwa et al. 2016)
Resistance to rice yellow mottle virus (RYMV)	Resistance to RYMV was introduced into IR64 background by marker assisted backcrossing.	(Ahmadi et al. 2001)

IR64 is generally considered a restorer line for hybrid rice, particularly in the WA CMS system widely used in *indica* “three-line” hybrid breeding (Xie et al. 2014). However, Toriyama and Kazama (2016) developed a new IR64 cms termed CW type (Chinese wild rice), restored by the *Rf17* gene.

At IRRI, induced mutation was used to generate a large collection of mutants in IR64. This collection has been used to discover many genes controlling important traits in rice (Wu et al. 2005). Some of these mutants show favorable phenotypes that could be used directly in rice breeding. Examples include tolerance to salinity (Nakhoda et al. 2012), resistance to blast disease (Madamba et al. 2009), resistance to tungro disease (Zenna et al. 2008), and drought tolerance (Cairns et al. 2009).

Transgenic applications have been limited for rice and are currently not in commercial cultivation. However, *Agrobacterium* mediated transformation protocols are well developed and used for IR64 (Ignacimuthu and Raveendar 2011). For example, it has been transformed with genes conferring higher Fe and Zn content in the endosperm (Oliva et al. 2014; Trijatmiko et al. 2016) and herbicide tolerance (Chhapekar et al. 2015).

## Conclusions

This brief review attempts to document the value of the variety IR64 for rice breeding and genetics but cannot contain all the information available. At one time, this variety was estimated to be grown on 9–10 million ha annually (Laird and Kate 1999). Considering the many years it has been in production, over approximately two decades, it has been providing hundreds of millions of consumers with high-quality rice. In this sense it resembles some of the other mega varieties like Swarna and Samba Mahsuri, grown in India. However, breeders have been quick to take advantage of this variety to make further advances. As an example, IR64 has been replaced in most of the Philippines and Indonesia by new varieties with similar quality attributes but improved agronomic traits like disease resistance and higher grain yield.

Rice breeding is a continual process and all varieties are expected to be replaced by improved varieties over time. IR64 is still a popular variety in some areas, particularly in India where it is a popular variety in the north. Its area is gradually declining, due to release of improved varieties. However, as outlined here, it lives on through its progeny that are cultivated or under evaluation throughout the region. Some of the most important factors that enabled the development of such a superior variety include: a large breeding program where many crosses were made annually and large segregating populations were grown, well-defined objectives with focus on the most necessary traits including preferred quality attributes, systematic screening by skilled researchers for required traits, sensory data to confirm quality of the cooked rice, a suitable evaluation program to measure yield as early in the breeding program as possible, and an effective outreach effort to evaluate advanced selections under farmers’ conditions and ensure that seed was widely available.

The development of high-quality mega varieties like IR64 has provided a challenge to rice breeders to make further improvements to varieties that are widely-accepted by farmers. This challenge has been met well in the Philippines and Indonesia where new varieties have replaced IR64, but less well in India where the variety is still popular. In general, new varieties that aim to replace the mega varieties must offer a clear advantage, such as improved stress tolerance or higher yield. Additionally, the rice processing industry must be supportive of the efforts to replace existing varieties with new ones, so that farmers will have a suitable market for their crop produced from these new varieties.

## Abbreviations

BB: Bacterial blight
BPH: Brown Planthopper
CSSL: Chromosome segment substitution library
GEU: Genetic Evaluation and Utilization
GLH: Green leafhopper
GT: Gelatinization temperature
HYV: High-yielding variety
IRRI: International Rice Research Institute
MABC: Marker assisted backcrossing
RIL: Recombinant inbred population
RTBV: Rice tungro bacilliform virus
RTSV: Rice tungro spherical virus
RVA: Rapid visco analyzer
WBPH: White backed planthopper
